# Everybody's Page

**Published:** 1890-11-22

**Authors:** 


### EVERYBODY'S PAGE.

A stickleback would not appear to be a very formidable sentinel. Yet the male fish keeps guard with success over the eggs during the process of hatching in the neatly-made nest made for the purpose out of any loose matters in running water, such as bits of grass or weed. Greedy enemies make constant raids on the stickleback's home, but like the proverbial weasel, he seems never to be caught napping.

---

Bald-headed men are beginning to look forward to a happy consummation. Dr. P. A. Morrow, at a recent meeting of the New York Academy of Medicine, gave the results of some successful experiments he has made in transplanting hair. He omitted to state at what season of the year hair-gardening should be done. Nevertheless, both old and youthful beaux who have suffered a premature loss of their ravishing locks are jubilant at the news.

---

An evident proof of the fact that smoking is deleterious to the juvenile occurred the last week in October in New York city. The *New York Medical Record* reports two cases of death, the result of smoking: the first, that of a boy "in whom heart disease had been superinduced by excessive use of cigarettes"; the other of an inveterate smoker aged sixteen, who hung himself because his father refused him any more money to spend on tobacco. It is quite probable that this boy was the victim of the nicotin insanity lately described by Kjelberg.

The Jews in Paris have a great number of charitable institutions, including a few asylums and refuges which are set apart solely for people of Hebrew persuasion, a hospital with nearly 300 beds, 40 mutual relief societies, of which 30 are for men and 10 for women, and numerous associations for the burial of the poor. Other nations might copy the example set them by the Jews, who invariably in all countries see to the wants of their poorer brethren.

---

A good cook is one of those luxuries often looked for but seldom obtained. If it is so hard to find this class of servant in town or country with the required attainments, it cannot be a matter of surprise that there is a scarcity of good plain cooks at sea. Wholesome cooking on board ships is a matter of no small importance; if the health and comfort of the crew are not what they should be, the result is almost certain to be discontent and accompanying want of discipline. The Shipmasters' Society have been moved to circularise the governing bodies of industrial schools and training ships on the subject. The suggestion that marine cooking classes should be established in these institutions is well worthy of consideration. The description of ship's tea as "water bewitched and tea betrayed" is, generally speaking, accurate, but this is really not always the fault of the cook. We fancy a good deal of the so-called tea that is sold is itself very much bewitched.

